# Does “Low Cost” Urban Sanitation Exist? Lessons from a Global Data Set

**DOI:** 10.1021/acs.est.3c05731

**Published:** 2023-11-03

**Authors:** Jin Igarashi, Barbara Evans, Andrew Sleigh, Davies N. Tarkash, Ronoh Kennedy, Ruthie Rosenberg, Fiona Zakaria

**Affiliations:** † School of Civil Engineering, University of Leeds, Leeds LS2 9JT, U.K.; ‡ Bureau for Policy and Programme Support, United Nations Development Program, One United Nations Plaza, New York, New York 10017, United States; § Narok Water and Sewerage Services Co., Ltd., P.O. Box 935-20500, Narok 935-2050, Kenya; ∥ Citywise Advisory Services, c/o Sanergy collaborative, Sameer Africa, Enterprise Road Nairobi, Nairobi 30429, Kenya

**Keywords:** urban, sanitation, cost, costing standards, benchmarking

## Abstract

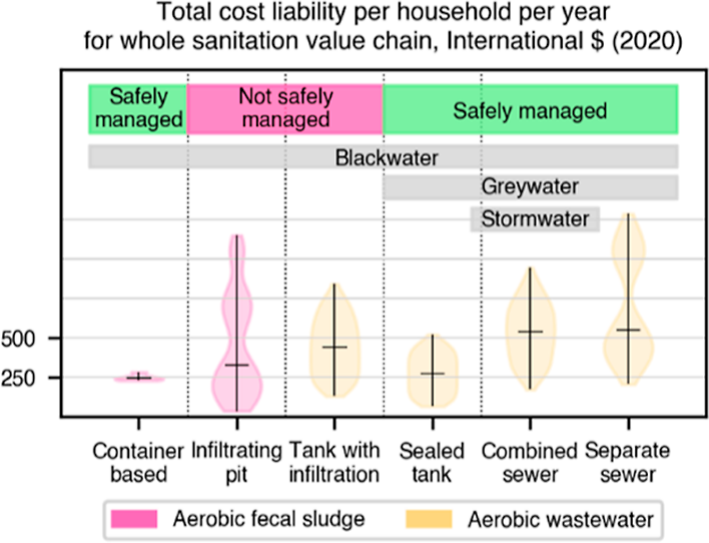

In this paper, we report results from, and demonstrate the value of, a global database for the collection and aggregation of reliable and comparable cost data for urban sanitation systems as they are built and operated on the ground (rather than the “as planned” costs that are often reported). We show that no particular “mode” of urban sanitation (for example “sewered sanitation” or “fecal sludge management”) can be meaningfully described as “low cost” when compared to other modes. We show that economies of scale may operate for systems that transport waste from pits and sealed tanks by road as well as for sewerage. We use a case study example to show the value of being able to compare local costs to global benchmarks and identify that operational considerations such as low connection rates may be more significant in determining overall cost liabilities for urban sanitation than technical considerations such as population density, size, and degree of centralization/decentralization.

## Abstract 2

Globally comparable quality cost data can improve planning and operation for safely managed sanitation in cities. Poor service coverage drives up the real costs of sanitation. Technology selection is less important than good management.

## Introduction

Nearly half (46%) of the 7.8 billion global population do not have access to safely managed sanitation;^1^ substantial investment is required to meet sustainable development goal (SDG) 6.2 of universal access. Contamination from ill-managed sanitation has significant negative impacts on public health and the environment, particularly in rapidly growing cities, and climate change will exacerbate these as it increasingly threatens the resilience of sanitation infrastructure and services.^2^ Policymakers and utilities thus face pressure to urgently evaluate the effectiveness of existing sanitation systems and efficiently plan for new ones. The vocabulary of “low cost sanitation” has been prevalent in the development discourse for many decades.^3,4^ Simultaneously, lack of reliable cost data for urban sanitation has been noted as a serious constraint to planning of sustained interventions globally.^5−8^ Dodane et al. (2012)^9^ were hampered in their efforts to understand the efficiency of price allocation between private and public actors by the lack of reliable international benchmark data on total costs.

Earlier work on costing has tended to focus only on certain, often highly engineered, system elements such as specific wastewater treatment processes, while many cost estimates are prospective and therefore highly prone to errors of underestimation. By contrast, the CACTUS project has developed a method to collect reliable comparable cost data for sanitation systems over different years and in different countries.^10^ The project has already collected comparable and reliable real cost data in 25 cities in 10 countries across the world as of June 2023.^11^ In this paper, we demonstrate the value of being able to sum the full costs of sanitation including collection, transport, and treatment and to examine these for the type of mixed sewer/onsite containment systems which are prevalent in African cities.

The cost of sanitation has been theoretically linked to a number of technical considerations such as population density, size, and degree of centralization.^8,10,12^ Here, we examine the evidence for other effects in determining the overall cost liabilities for urban sanitation.

The CACTUS database can be used to establish global cost estimates, and it can also be used to better understand costs and cost drivers in single-city locations. This paper demonstrates this approach, using data from a city in Kenya, as an example of how global benchmarking comparisons can help to reveal the costs and cost drivers of sanitation in a single city.

## Materials and Methods

In this study, costs were organized using the CACTUS method described by Sainati et al.^10^ The CACTUS project classifies sanitation systems using 27 “component categories” across the entire sanitation value chain. Component categories allow for both benchmarking of costs between systems using similar components and for the construction of cost estimates for complete sanitation systems, even in cases where all elements of the sanitation value chain are not in place. These are summarized in Supporting Information Figure S1. Detailed descriptors for each component are available in the data collection manuals on the CACTUS Web site.

CACTUS also uses a set of consistent and comprehensive cost categories (Supporting Information Table S1). A cost item is categorized as either capital costs (CAPEX) or operational costs (OPEX). Generally, costs with a lifetime or replacement period of less than 1 year are categorized as OPEX. Both CAPEX and the OPEX costs are further disaggregated into direct costs (expenditure which is required to buy, build, or purchase goods and services required to construct and operate the system) and indirect costs, required to manage the process (typically staff costs, management, human resources, insurance, legal, and financial services). To avoid double-counting, any payment that moves as a fee between the operator of one component and the operator of another component is excluded. Thus, for example, fees paid by households for emptying pits and tanks are not counted as the OPEX for containment. The real cost of emptying (rather than the fee income) is accounted for in the “emptying” or “emptying and transport” element of the sanitation value chain. For containments, OPEXs are limited to anything that must be done to maintain the infrastructure or keep the facilities clean.

CACTUS uses the costs of existing operational systems and not projected or planned costs. Operational factors such as emptying frequency for infiltration pits and sealed tanks are not assumed but are as reported in the operators’ data. The total number of households served is either reported (for example, when the number of connections to a sewer network is known) or calculated (for example, based on emptying frequency and the numbers of emptying events completed in an annual cycle for emptying and transport operators). The costing approach is set out in Sainati et al. (2020).^10^

Data are assembled by researchers working in selected case study locations. The case studies to date have been selected pragmatically from partners who are motivated and willing to participate in the project. Data collection typically takes place through a series of workshops which bring together key stakeholders and one-to-one meetings (usually with staff from the accountancy department and/or the Chief Financial Operating Officer) supplemented by inspection of primary and secondary data sources (accounts). The workshops help to build an understanding of the need for and methods used to assemble comprehensive estimates of the real costs of sanitation service delivery. In some cases where service providers are acting relatively independently (for example, some private sector pit emptying services), data can be collected through interviews and inspection of records without the need for a workshop.

Data are summarized on standard workbooks downloaded from the CACTUS Web site. Once checked and verified for completeness and internal consistency, these are shared with key informants prior to being uploaded to the CACTUS database. CACTUS data are processed, so that raw cost information can be normalized for comparison purposes. Results are expressed in International Dollars, the equivalent year 2020, and have been updated to a new comparison year and with new data points since the publication of Sainati et al. (2020).^10^

CACTUS uses two cost indicators: the total annualized cost per household (TACH) and the total annualized cost per capita (TACC). Both TACH and TACC include annual OPEX plus the annualized cost liability associated with covering the CAPEX for the system over its lifetime. It is thus a full-costing approach^13^ and the results can be used for capital budgeting.

The total cost liabilities for theoretical “complete” sanitation systems are generated by CACTUS using the data on partial systems collected in the case studies. Archetypal systems are created by combining cost data for only those components that can technically be combined (for example, direct connections, with one type of sewerage and one type of wastewater treatment, or sealed tanks with mechanical emptying and transport and one type of fecal sludge or one type of wastewater treatment). Thus, each archetypal system comprises either 3 or 4 components. A list of archetypal systems for which CACTUS currently has data is shown in Supporting Information Table S2. Cartesian products are generated by combining the extracted TACH values for each component. The full lifecycle (TACH) costs are then generated for the archetypal system by summing those for components and filtered for the interquartile range. The results are rounded and plotted using violin plots. We selected 12 illustrative archetypal systems for further analysis. These were selected by first sorting on the type of containment and then, within each group, selecting the systems with the highest number of data points available from which we could construct synthetic estimates. In all cases, at least 20 data points were available, with the exception of the container category (see Supporting Information Table S2).

Having reported global results from the CACTUS database, in the results below, we also use the specific example of Narok town in western Kenya to demonstrate how CACTUS data can be used at the local level to examine cost drivers. Fieldwork in Narok, a town with a population of just over 100,000 and around 30,000 households^14^ yielded data for a total of 30 cost data points and 6 components across most elements of the sanitation value chain. The results were used to benchmark costs in Narok against those in the global database.

## Results and Discussion

**Figure 1:**
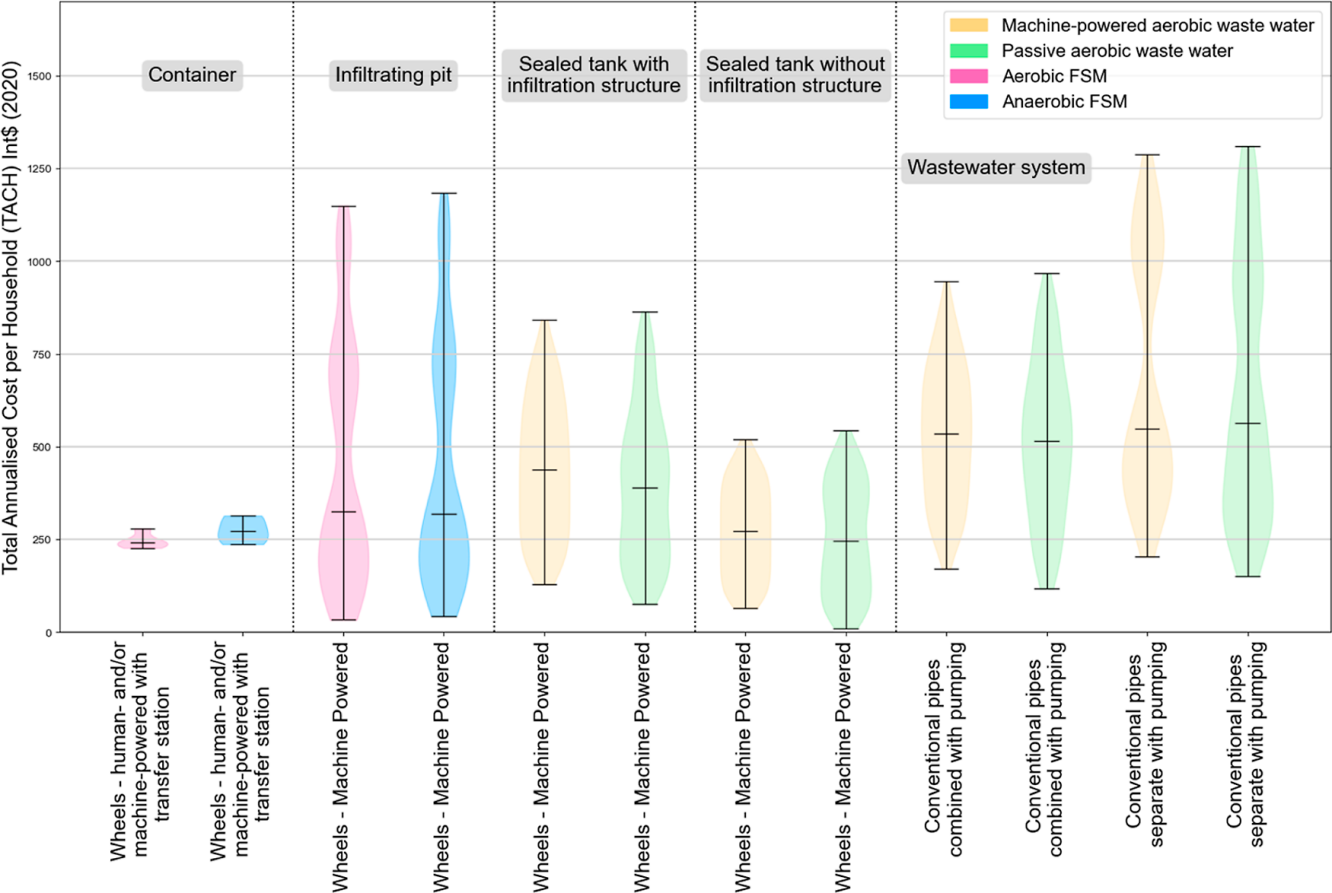
Distribution of summed total annualized cost liability [Int$ (2020)] for synthetic archetypal urban sanitation systems, based on 125 data points collected by the CACTUS project as of June 2023. The horizontal bar shows the median cost for each system. Sealed tanks without infiltration which are regularly emptied can be used to manage both black and gray water safely while pits and tanks with infiltration cannot and will therefore be unsuitable in most urban places. All sewers carry domestic gray water along with blackwater; combined sewers also carry stormwater. Equivalent cost effectiveness cannot therefore be inferred across all systems based on costs alone.

As of June 2023, the CACTUS database contained 125 data points from ten countries (Bangladesh, China, Ghana, Guyana, India, Kenya, Peru, Senegal, Thailand, and Zambia). The distribution of data points is summarized in Supporting Information Table S3. Each data point represents costs borne by a single service provider for delivery of an individual component of the sanitation value chain. For containers such as infiltrating pits or sealed tanks, one data point may therefore be the costs reported for a single containment by a single household (when self-financing) or costs borne by a program to deliver and operate multiple containments. Summary statistics including median and mean TACH and TACC along with CAPEX and OPEX data for each component are shown in Table 1. Synthesized estimates for the total annual cost liabilities for 12 archetypal sanitation systems generated from the data available in the CACTUS database are in Figure 1.

**Table 1:** Summary Cost Liabilities for Typical Components of Urban Sanitation Derived from the CACTUS Database as of June 2023 on a per Household Basis in Int$ (2020)

element/component^a^	number of data points(*n*)	total annualized cost per household served - TACH median (mean)	total CAPEX per household served median (mean)	annual OPEX per household served median (mean)
**containment**	50			
container	2	**127** (127)	**190** (190)	**74** (74)
direct (connection to sewer)	8	**118** (149)	**1547** (1604)	**0** (25)
infiltrating pit	21	**139** (412)	**1227** (5789)	**28** (62)
sealed tank with infiltration structure	10	**191** (473)	**1574** (5845)	**102** (125)
sealed tank without infiltration structure	9	**71** (83)	**637** (1013)	**0** (16)
**emptying**	4			
human-powered with specialized equipment	2	**33** (33)	**1** (1)	**32** (32)
manual (no specialized equipment)	2	**80** (80)	**15** (15)	**76** (76)
**emptying and transport**	35			
**pipes (sewers)**				
conventional, combined, with pumping	7	**262** (269)	**2862** (2678)	**34** (29)
conventional, separate, no pumping^b^	2	**3279** (3279)	**52,566** (52,566)	**295** (295)
conventional, separate, with pumping	6	**198** (379)	**2875** (4520)	**68** (94)
**wheels (trucks)**				
human- and/or machine-powered with a transfer station	1	**101** (101)	**4** (4)	**100** (100)
human-powered	2	**147** (147)	**20** (20)	**145** (145)
machine-powered	17	**27** (40)	**46** (89)	**19** (29)
**transport**	2			
**wheels (trucks)**				
human- and/or machine-powered with a transfer station (transport only)	1	**1** (1)	**6** (6)	**1** (1)
machine-powered (transport only)	1	**23** (23)	**46** (46)	**16** (16)
**treatment**	34			
aerobic FSM	6	**16** (30)	**41** (94)	**10** (23)
anaerobic FSM	3	**44** (46)	**361** (502)	**18** (16)
aerobic wastewater	1	**146** (146)	**1916** (1916)	**14** (14)
machine-powered aerobic wastewater	15	**132** (156)	**1558** (1688)	**50** (50)
passive aerobic wastewater	9	**38** (124)	**58** (1219)	**9** (53)

a Components for which there are zero data points are not shown.

b Data point contains Narok outlier (see below).

These archetypal systems are all models of complete “sanitation value chains”, but they are not directly comparable in terms of the level of service provided, even when properly designed, constructed, and operated. Combined sewers generally provide a higher level of service to the household because they carry all domestic wastewater (including both fecal waste and domestic gray water) as well as stormwater to treatment. Some of the mixed flow may be diverted through combined sewer overflows during rainfall events. Separate sewers also carry all domestic wastewater, but not stormwater. Sealed tanks with infiltration structures (a category which sometimes but not always includes true “septic tanks”) with emptying and transport may enable conveyance of a portion of household fecal waste and domestic wastewater to treatment in the form of sludge emptied from the tank. However, some of the liquid fractions including fecal matter will infiltrate the ground or, more probably in urban areas, contaminate surface water bodies via overflow pipes. Where there is no infiltrating pit attached to a sealed tank, the liquid fraction can flow out into surface water bodies only via an overflow pipe. Infiltrating pits (sometimes referred to as pit latrines) when properly managed with manual emptying and aerobic fecal sludge treatment will convey most household fecal waste to treatment when properly managed but liquid fractions including household gray water and some fecal matter will be infiltrated or more commonly diverted to surface water bodies. Containers capture household fecal waste and sometimes gray water. None of the latter four categories has the potential to convey any stormwater.

Total annual cost liabilities in these systems lie broadly within comparable bounds but with high levels of variation within the results for each system driven by local context and how well systems are built and operated. Our data set currently contains information on two cases of container-based sanitation (CBS) providing shared toilets, whose median total cost liability per household is around Int$ 250 (2020). Although the removable containers used in CBS have a shorter lifespan than “concrete” containment (pits and tanks), their provision includes the toilet pan/seat and often a superstructure. Taking lifespan and total costs into account, CBS with well-managed emptying and treatment may be highly cost-efficient compared to other comparable services that do not convey stormwater. Scale effects may drive some of this cost efficiency (discussed below). Since the number of data points is very small, this result requires further investigation. The median costs for all the other archetypal systems for which we have calculated costs, all lie between Int$ 350 and 550 (2020). Some forms of sewers and mechanical and manually emptied sanitation systems, all appear to have broadly comparable overall costs.

While this conclusion should be treated with caution due to the relatively small size of the database and the paucity of data for some components of the sanitation system, it suggests that the concept that some technical approaches to urban sanitation provision are inherently “low cost” compared to others may have no evidential basis.

**Figure 2:**
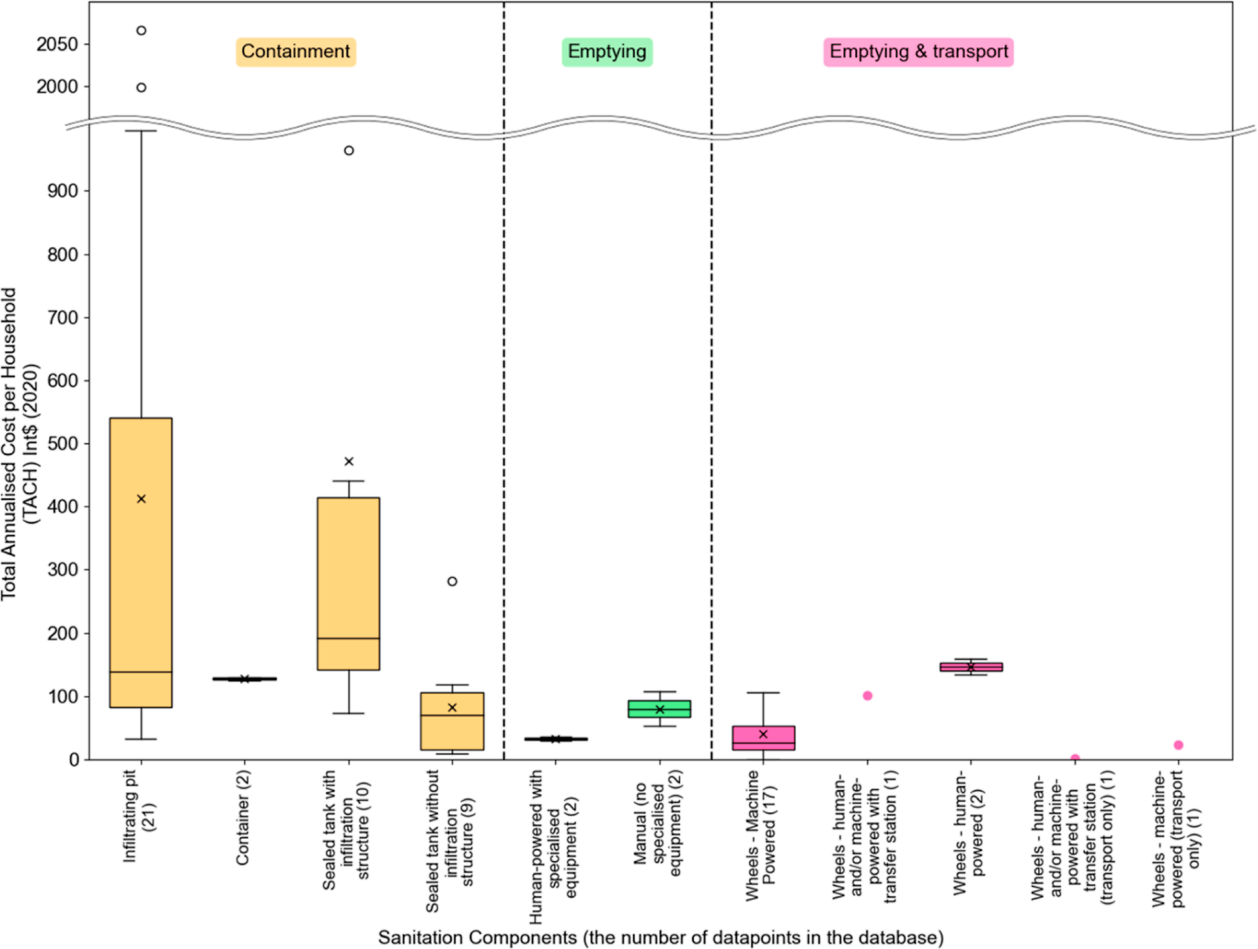
Total annualized cost liability [Int$ (2020)] for containers and emptying/transport in urban sanitation systems with no sewerage from data collected by the CACTUS project as of June 2023. Boxes show the interquartile range, *x* is the mean, and the horizontal bar is the median for each component.

As well as the costs for composite archetypal systems, CACTUS data can be interrogated for information about specific components and their costs. Turning specifically to systems which do not use sewers to convey any fecal flows, containment systems (for example, lined, sealed, or infiltrating pits and tanks but excluding direct connections to sewers) vary in cost between Int$ 9 and 2139 (2020) (Figures 2 and Supporting Information S2A). Emptying services vary in price between Int$ < 1 and 159 (2020).

Mean values for containment are high relative to the mean costs for emptying, meaning that in some contexts there is a relatively small contribution of emptying and transport to overall cost liabilities (summarized graphically in Supporting Information Figure S3).

The costs per household of onsite containment (both infiltrating pits and sealed tanks with infiltration structures) vary significantly due to both CAPEX (largely a function of the scale and design features of the toilet) and the intensity of usage. The highest overall cost liabilities are associated, unsurprisingly, with larger and more elaborate structures. These large structures are often private and sometimes carry a cost liability of tens of thousands of dollars. The three most costly systems in the database are all private toilet and bathroom complexes serving single families or small family groups.

Somewhat counterintuitively, the costs of manual emptying and transport tend to be higher than those for mechanized systems (Figure 2); the mean cost for cases of manual emptying in our data set is more than 3.5 times higher than the average cost of the mechanized systems. This difference is largely driven by time and cost of labor. Manual emptying is often unregulated and may result in additional health and social burdens on operators.

**Figure 3:**
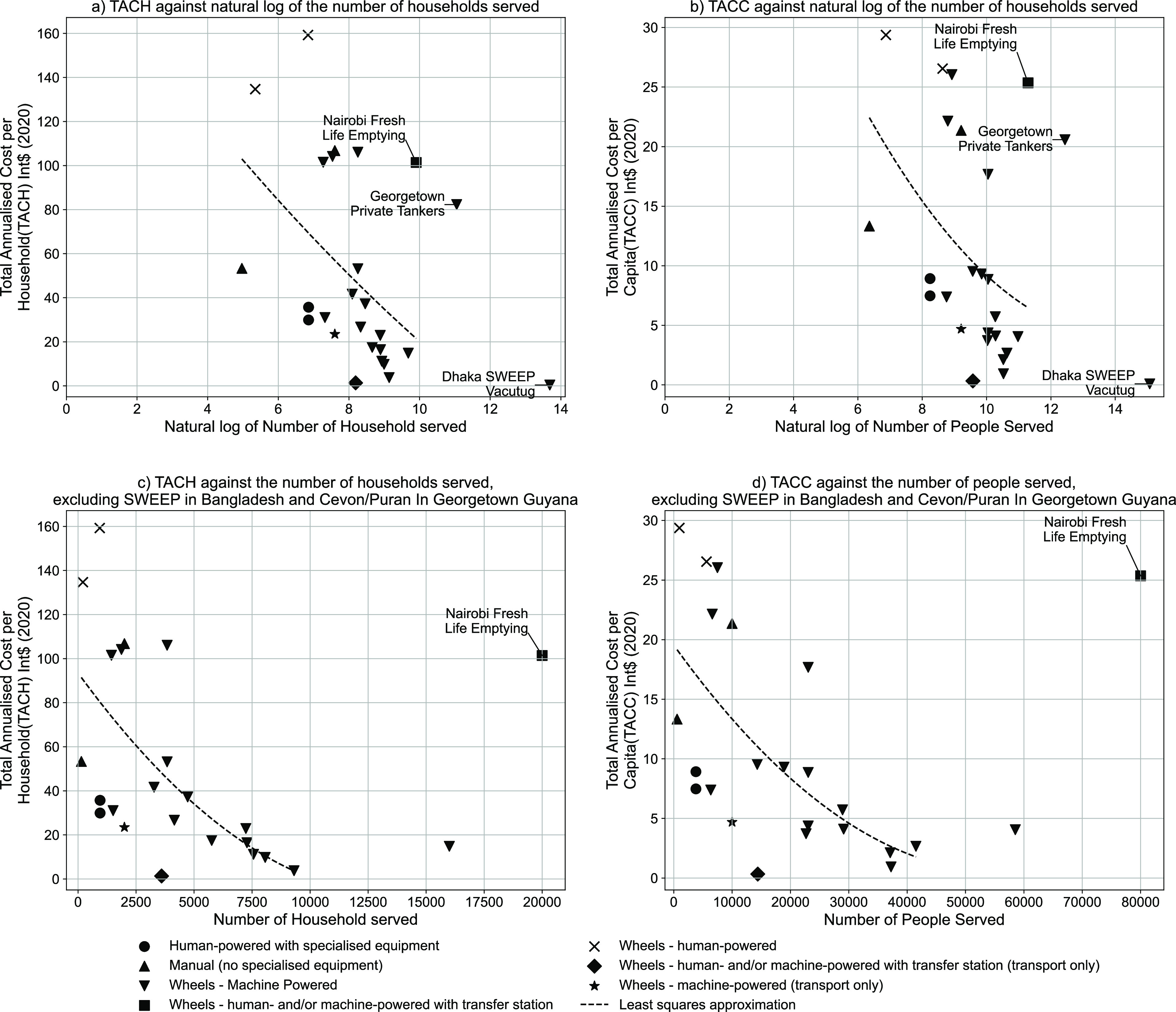
Total annualized cost liability [Int$ (2020)] for emptying and transport services from data collected by the CACTUS project as of June 2023. Best fit lines by least-squares method, after removal of outliers (quantile less than 0.95).

To examine the effect of scale on emptying and transport operations, we plotted the TACH and TACC of all mechanical emptying and transport services against the natural log of the total number of households served (Figure 3a,b). There is an apparent relationship between larger-scale systems associated with lower costs. There are four large-scale service providers in our data set (SWEEP in Bangladesh, two private operators in Georgetown, Guyana, and Fresh Life Toilets in Nairobi). These services operate at a scale which is in an order of magnitude greater than most of the cases included in our data set. After removing SWEEP and the two data points from Guyana, it is possible to examine the non-logarithmic relationship between cost and scale for the remaining smaller operations (Figure 3c,d). For this set of providers, there is a general downward trend in TACH and TACC as the scale increases. As scale increases, the efficiency of the operation rises, since certain costs (for premises, management, and so on) are fixed and largely independent of the number of households served. At a certain scale, the costs appear to rise significantly (note the Fresh Life data point in Figure 3c,d), and then inspecting Figure 3a,b scale again seems to be associated with a downward trend in TACH and TACC. Inspection of these three data points shows a higher proportion of expenditure on indirect costs including insurance, staff training, health protections and management, and the payment of regulatory fees and/or taxes. All of these are costs that could arguably be required for all service providers to meet basic minimum standards for health, safety, and social development. It also suggests that these larger operators are taking on the coordination and management roles that may be provided by the state/local authorities when small-scale providers are contracted or otherwise allowed to operate. These coordination costs (for example, to manage call centers, or administer operational contracts for smaller providers) are all part of the overall costs of service delivery, suggesting that for smaller operators reporting only their own costs, the estimates may be artificially depressed.

Despite the higher costs of the CBS emptying and transport services in our data, the overall costs of CBS remain relatively low. The lowest overall cost liabilities appear to be associated with systems that are regularly emptied (including containers in CBS systems and sealed tanks without overflows, which are regularly emptied).

**Figure 4:**
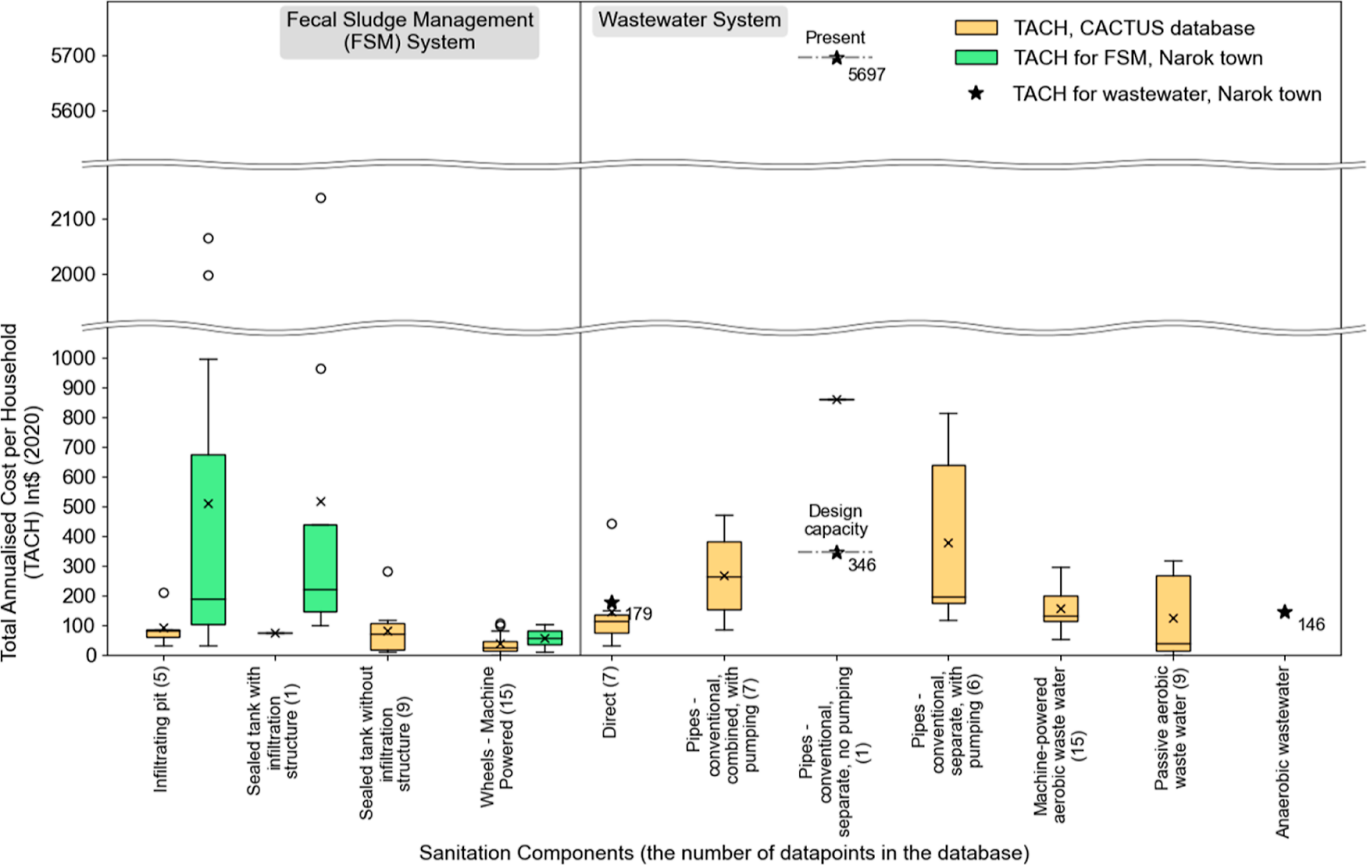
TACH [Int$ (2020)] by component for Narok town (mean values shown as star icons), compared to the CACTUS database (full data range shown excluding data from Narok) as of June 2022. Boxes show the interquartile range, *x* is the mean, and the horizontal bar shows the median for each component. For the sewerage (pipes-conventional, separate, no pumping) in Narok, the current value of TACH and the theoretical value of TACH assuming 100% design connectivity is achieved are both shown.

CACTUS can generate globally informative estimates of costs but can also be used to examine cost outcomes in a specific city or town. Here, we use the town of Narok in Kenya to illustrate the point. Just over 80% of people in Narok have sanitation services delivered through “onsite” pits and tanks with road-based emptying services.^15^ Three percent have access to sewers, and some open defecation still takes place. Around 60% of excreta are safely managed, of which just over half is stored and never emptied from onsite containment with most of the balance transported from containment to the treatment plant. There is a newly constructed sewer network. Cost data for a total of 30 sanitation components have been collected for Narok and these can readily be compared to data in the global database (Figure 4 and Supporting Information Table S4).

The cost profiles for the wastewater system in Narok are dominated by low connection rates to the new sewer network (which is operating at 6% of its design capacity and serves only 2% of the total population). Households that have onsite pits or tanks have made significant investments in their existing sanitation infrastructure. TACH for onsite containment in Narok is largely in line with the global data set, although there are some much more costly systems, often within private households. Onsite containment costs per household in Narok are broadly inversely related to the number of households or people served (see Supporting Information Figure S5). There are some outliers, with significantly higher TACH/TACC values; these are often extremely large and well-built and used by individual private households. There may also be some inefficiencies in the costs of emptying and transport services (Supporting Information Figure S6).

The current policy framework is based on the expectation that the full cost of direct connections to sewers should be passed on to households. However, it appears that households have a limited incentive to switch to sewer connections.

**Figure 5:**
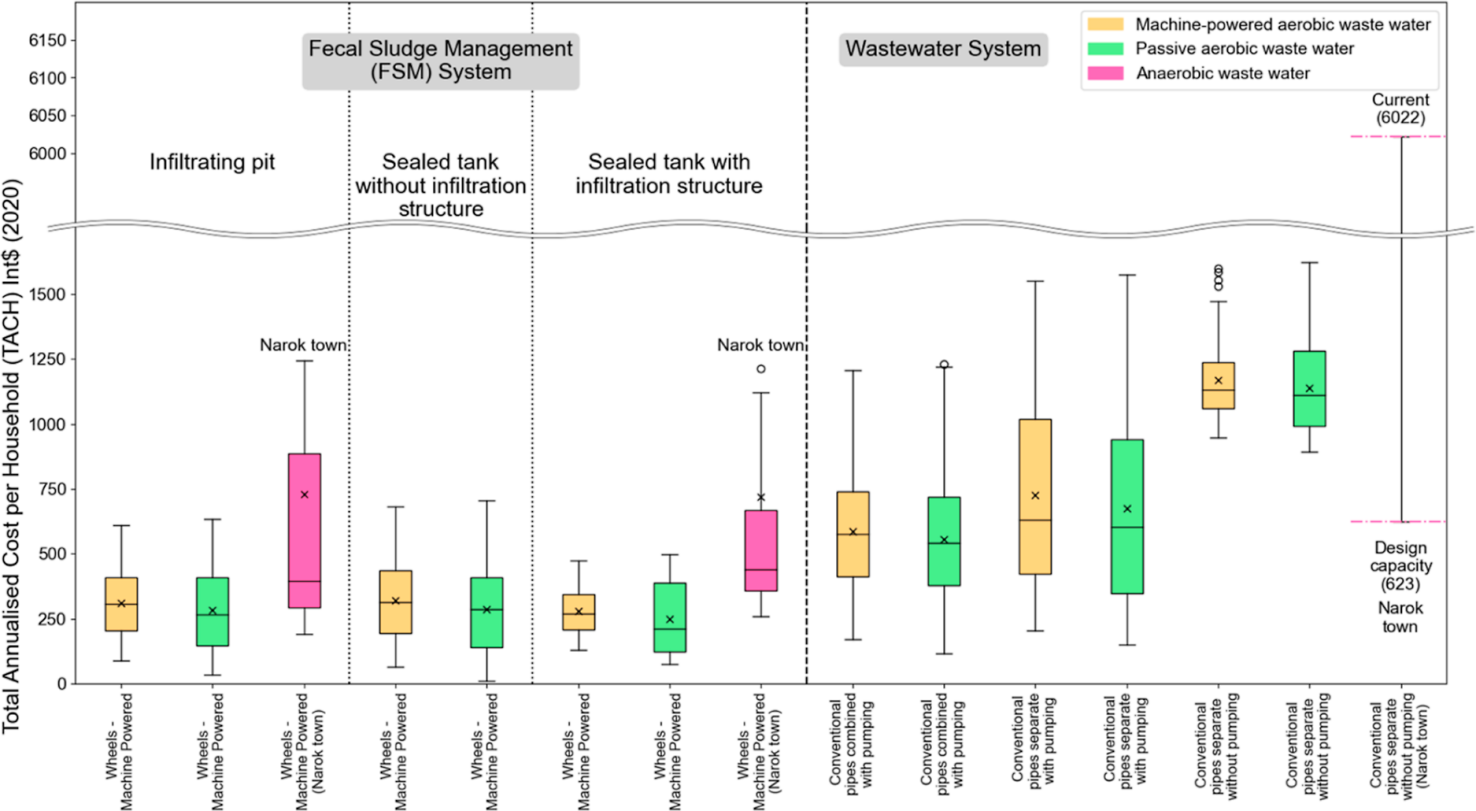
TACH [Int$ (2020)] for whole sanitation systems for Narok town (box plots) compared to the CACTUS database (bars), excluding the data from Narok, as of June 2022. Boxes show the interquartile range, *x* shows the mean, and the horizontal bar shows the median for each component.

Figure 5 compares the full-system TACH of existing sanitation systems in Narok town to full-system TACH estimates generated from the CACTUS database. The cost liability per household for wastewater services in Narok is exceptionally high as a result of the low connectivity rate.

**Figure 6:**
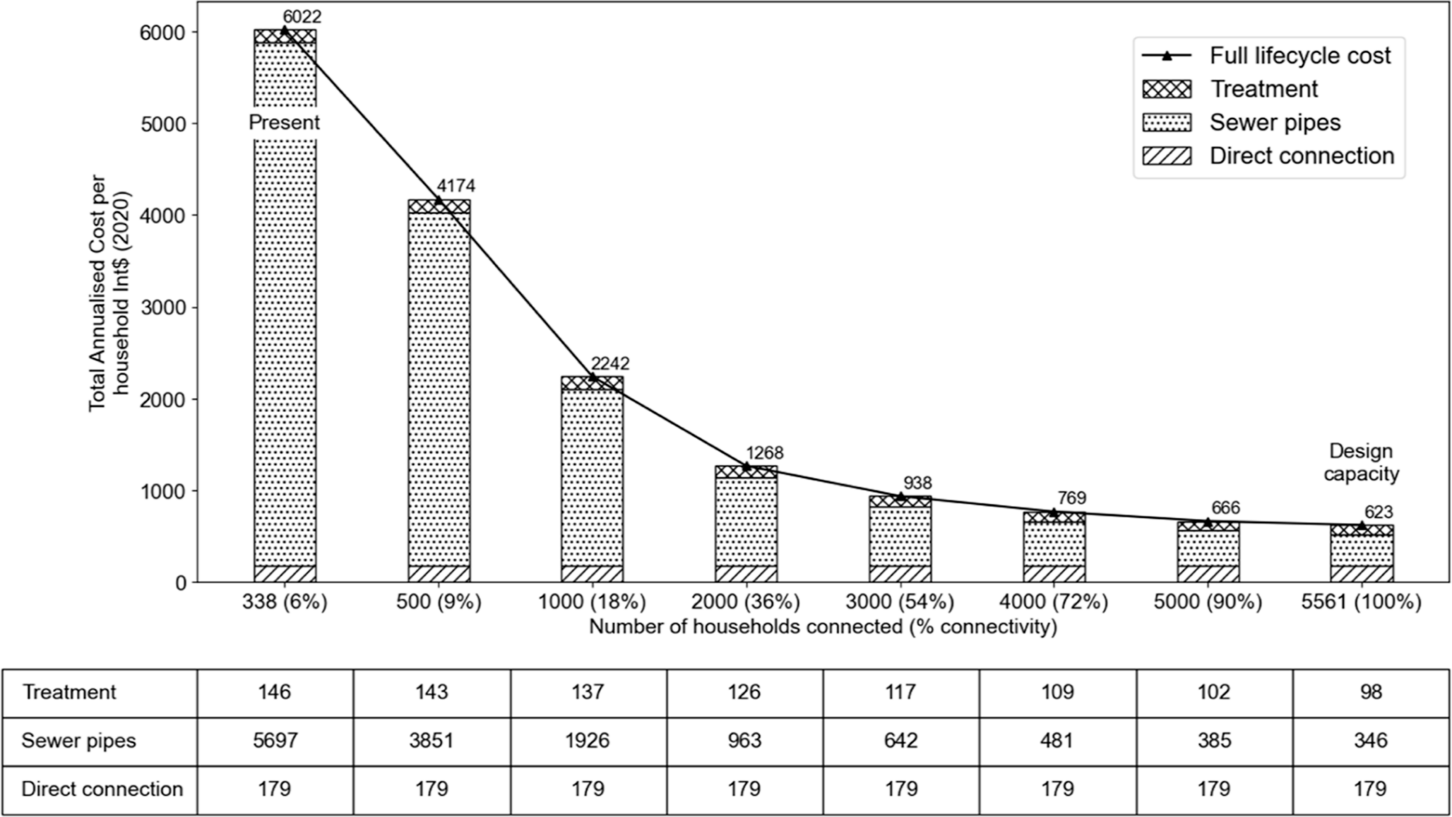
Whole-system TACH [Int$ (2020)] of sewerage for Narok town plotted against sewer connectivity. The stacked bars illustrate the share of the costs associated with each element of the sanitation value chain, and the line chart shows the total cost plotted against percentage sewer connectivity. The data table shows TACH broken down by component, corresponding to the bars above.

Economies of scale might be expected in sewered sanitation systems relating both to the extent of the system and the number of households within the service area.^16−19^ However, these scale effects are indiscernible in Narok. The whole-system TACH for sewerage could be reduced by almost an order of magnitude to Int$ 623 (2020) if the system was running at full design capacity (Figure 6).

Around 90% of the whole-system TACH for the wastewater system in Narok comprises capital costs, both direct and indirect, and approximately 40% of the total cost comprises financing costs for a loan from the African Development Bank, plus taxes (Supporting Information Figure S4). While there is limited data on the costs incurred to finance sewer investments elsewhere,^9,20^ the CACTUS database^11^ includes data from Shenzhen, China, in which loans were used to finance machine-powered aerobic wastewater treatment, with the costs of borrowing comprising from 5.7 to 7.2% of total costs. While it could be argued that the loan is required to make the initial capital investment, the long-term impact on the overall cost liability is high in Narok.

The current accounting system in Narok suggests that operation and maintenance liabilities are around 10% of total costs for both sewers and wastewater treatment (Supporting Information Figure S4). This may be an underestimation, given the paucity of actual OPEX data available in Narok. CACTUS data from other cities show the share of total costs on operations at 30% for sewers in Nakuru, Kenya, 20% in Dakar, Senegal, and 32% in Lusaka, Zambia, and for treatment 41% in Dakar and 48% in Lusaka. This suggests that Narok town may be underestimating the long-term OPEX liabilities of their sewerage system.

These issues discussed above are not specific to Narok but reflect the real challenges of delivering high-quality sanitation in towns and cities.

## Implications

The CACTUS database and the use of TACH form the basis for cost comparisons for complete urban sanitation systems. Our analysis shows the value of a globally consistent database of comparable cost data for urban sanitation. Reporting the full cost liability of sanitation systems (which includes annualized CAPEX liabilities and ongoing annual OPEX liabilities) is useful in moving away from simple categorizations of “low cost” or “expensive” technology and would help utilities and local governments to plan and budget for delivery of SDG 6.2. Our results so far suggest that complete, well-managed, urban sanitation systems should typically result in an annual liability to the operator of between Int$ 250–550 (2020) per household, with higher costs associated with systems that convey gray water (and sometimes stormwater) in addition to household black water. The database provides no evidence that any of the current modes of sanitation service delivery could accurately be described as “low cost” compared to any other when their full functionality is taken into account. The small number of CBS services in our data set have lower overall cost liabilities compared to other systems that transport fecal waste by road but provide shared rather than household toilets.

Local conditions including both physical and market conditions undoubtedly have an impact on overall cost liabilities for urban sanitation, but the database is currently too small to support a very detailed analysis of these factors. However, from data currently available in the CACTUS data, the following conclusions relating to cost efficiency can be tentatively drawn.

First, our data suggest that where systems are completed and operate at the capacity for which they were designed, scale has benefits both for systems which empty and move sludge from containments by truck and for sewerage. Larger-scale operators report a relatively higher proportion of indirect costs, which are likely to be associated with activities that promote sustainability and equity, such as ensuring appropriate salaries and supervision of sanitation workers, investments in insurance, and payment of due taxes. This point can be extended to note that while manual emptying often has lower start-up (CAPEX) costs compared to mechanized emptying services, the long-term cost liabilities of manual emptying are often higher and result in less sustainable and equitable outcomes. Many of these operations remain at a small scale and have relatively high costs of time and labor.

Second, while scale is clearly a driver of cost efficiency, the actual completion of interventions, including connecting households to available services, may be much more significant. Incomplete systems which do not enable households to benefit from containment, emptying, transport, and treatment do little to move us toward global targets for safely managed sanitation, while still burdening local authorities or households with cost liabilities. This is clear from the results in Narok and applies equally to the piped and wheeled systems.

The overall costs of most complete well-managed sanitation systems are not insurmountable (averaging out at around Int$ 0.68–1.50 (2020) per household per day). A proportion of the total cost liability for many systems that store excreta at the household level and transport it by road is household containment. Where this investment has already been made (as is often the case in low- and middle-income cities and towns), the cost efficiency of providing a well-managed emptying, transport, and treatment service may be high.

Further data collection and an expansion of the data set would lend greater confidence to our conclusions. The data set currently is relatively small, and it is therefore challenging to know how to deal with outliers. A larger data set would enable a more confident and detailed analysis. Interrogation of a larger global data set might enable a better understanding of the cost implications of operational characteristics such as emptying frequency as well as contextural drivers such as population density, topography, and geographical location. The CACTUS database is an open-access resource including online systems for uploading data, with manuals and online support available. CACTUS can thus be used to structure the collection and analysis of urban sanitation cost data while contributing to the creation of an open-access global data set.

## Supporting Information Available

Sanitation components, cost elements, potential archetypal systems, distribution of total annualized cost per household per component, ranked total annualized cost per household for containment, emptying and treatment, mean modelled total annualized cost per household for archetypal systems, summary cost data from Narok, distribution of cost liability for sewerage in Narok, cost for emptying and transport against scale for Narok, distribution of cost liability for mechanical emptying and transport in Narok (PDF)
