# Supplementary material for: Does “Low Cost” Urban Sanitation Exist? Lessons from a Global Data Set
Source: Environ Sci Technol. 2023 Nov 3;57(45):17237–45. doi: 10.1021/acs.est.3c05731 (PMC10653215; doi:10.1021/acs.est.3c05731)
Supplement: Supplementary file 1 — es3c05731_si_001.pdf [file es3c05731_si_001.pdf]

Does 'low cost' urban sanitation exist? An examination of a global dataset

Jin Igarashi[1,2], Fiona Zakaria[1], Andrew Sleigh[1], Davies N. Tarkash[3], Ronoh Kennedy[3], Ruthie Rosenberg[4], Barbara Evans[*,1]

1/ School of Civil Engineering, University of Leeds, LEEDS, LS2 9JT, United Kingdom

2/ Bureau for Policy and Programme Support, United Nations Development Program, One United Nations Plaza, New York, NY, 10017, U.S.A

3/ Narok Water and Sewerage Services Co., Ltd., P.O Box 935-20500, Narok, Kenya

4/ Citywise Advisory Services

SUPPLEMENTARY INFORMATION

Summary: 15 Pages, 6 Figures, 4 Tables.

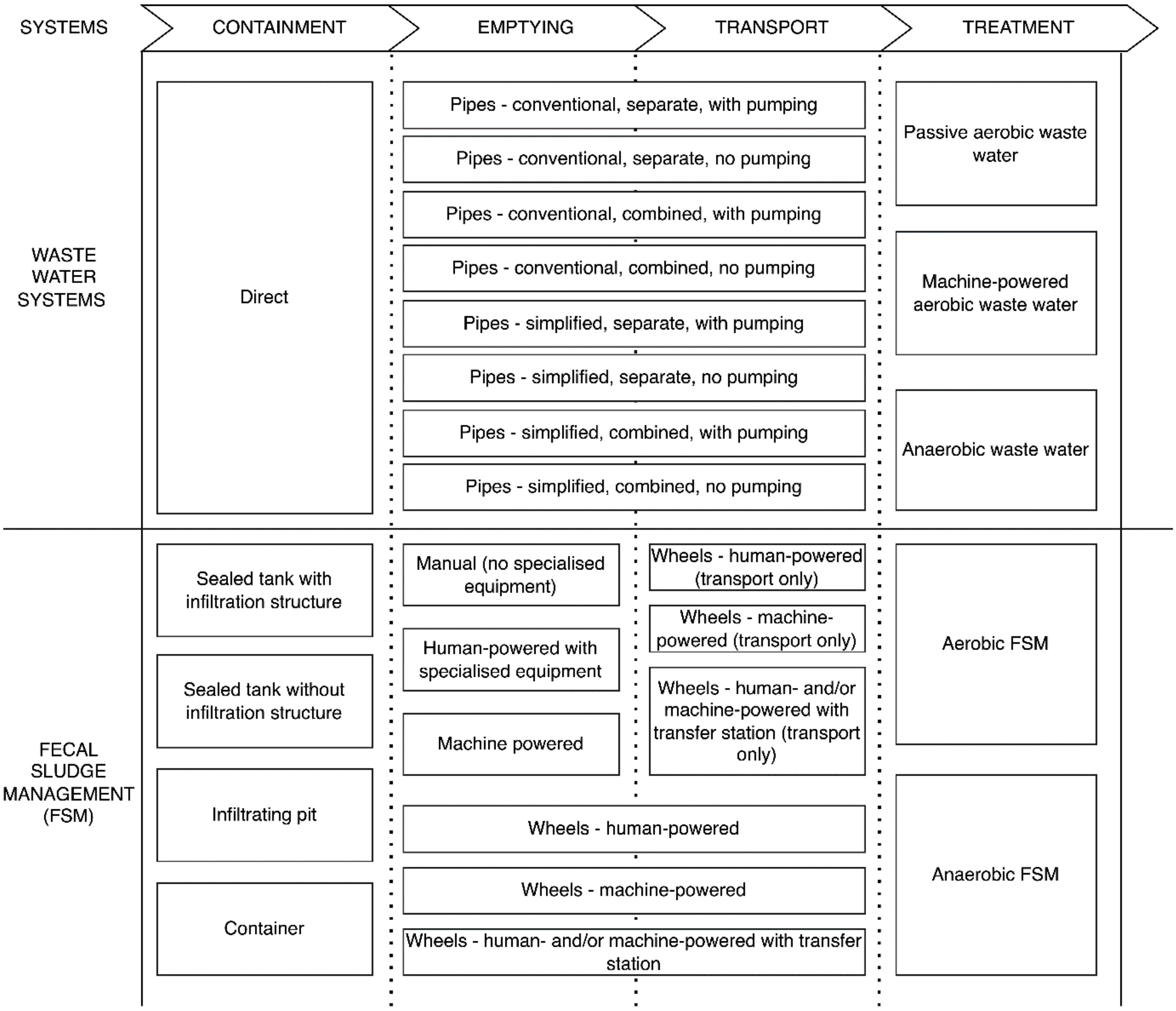

Figure S-1: Sanitation component categories used in the CACTUS database (reproduced and further developed from Sainati et al., 2022).

Notes on Figure S-1 (summarised from the CACTUS manuals):

1. Wastewater treatment plants sometimes also incorporate facilities for co-treatment of feacal sludge transported from sealed tanks or infiltrating pits. These are categorised as wastewater treatment components for the purposes of CACTUS analysis.
2. *Conventional sewers* use ‘traditional’ hydraulic design approaches and are usually laid under the road, flowing by gravity. Pumping, when it is included, usually occurs at pumping stations at the junction between two gravity flow sewers.
3. *Simplified sewers* use modified hydraulic design approaches, resulting in small diameter sewers laid at shallower slopes than conventional sewers. These are sometimes laid under sidewalks or properties resulting in shorter total lengths of sewers.
4. *Manual (no specialised equipment) emptying* - refers to the practice of digging or bucketing the contents of pits and tanks directly by hand using only buckets or shovels with no mechanical aids.

5. *Human-powered with specialised equipment emptying* – refers to the practice of manual emptying using non-motorised equipment such as the gulper, rammer, MDHP or MAPET.
6. *Machine-powered emptying* – refers to the practice of emptying pits or tanks using a motorised pump.
7. The three *transport only* categories are used to refer to the cases where waste that has previously been emptied using any one of the three emptying methods, is transferred into a container (tank or sealed barrels) mounted on a wheeled vehicle and moved by means of a hand cart, or truck.
8. The three *emptying and transport categories which use wheels* (to distinguish them from systems which use pipes) are used to refer to the case where the means of transport (tank or sealed barrels) is filled directly from the pit or tank, either manually or more commonly mechanically.
9. Transport including a *transfer station* refers to the case when pits or tanks are emptied and the contents are delivered to a holding tank away from the house. An operator then moves the waste on to treatment. Where a single operator manages both stages of this operation, costs may be reported in the combined category 'Wheels – human- or machine-powered with transfer station'. Where costs can be disaggregated for the emptying stage and the transport stage, they will be reported in the relevant separate categories.

Table S-1 Cost elements included in CACTUS data collection and reporting (direct and indirect)

**Capital costs (CAPEX)**	1. Land<br>2. Infrastructure and buildings<br>3. Equipment<br>4. Staff development<br>5. Major and extraordinary repairs<br>6. Other CAPEX<br>7. Administrative Charges<br>8. Financing<br>9. Taxes		
**Operational costs (OPEX)**	1. Land<br>2. Infrastructure and buildings<br>3. Equipment<br>4. Staffing<br>5. Consumables ------------	5.1 Utilities<br>5.2 Fuel<br>5.3 Chemicals<br>5.4 Services ------------<br><br><br><br><br>5.5 Other consumables	 <br><br>5.4.1 Consulting/ advisory<br>5.4.2 Legal<br>5.4.3 Insurance<br>5.4.4 Regular Maintenance<br>5.4.5 Other services
	6. Other OPEX<br>7. Administrative Charges<br>8. Financing<br>9. Taxes		

Table S-2: Potential Archetypal Sanitation Systems based on Datapoints in the CACTUS database at June 2023 (n= number of datapoints per category), lines in bold italics are shown in Figure 1

Contain-ment	n	Emptying	n	Transport	n	Emptying & Transport	n	Treatment	n	Total n
Container	***2***					***Wheels - human- and/or machine-powered with transfer station***	***1***	***Aerobic FSM***	***6***	***9***
Container	***2***					***Wheels - human- and/or machine-powered with transfer station***	***1***	***Anaerobic FSM***	***3***	***6***
Infiltrating pit	***21***					***Wheels - machine powered***	***17***	***Aerobic FSM***	***6***	***44***
Infiltrating pit	***21***					***Wheels - machine powered***	***17***	***Anaerobic FSM***	***3***	***41***
Infiltrating pit	21	Manual (no specialised equipment)	2	Wheels - human- and/or machine-powered with	1			Aerobic FSM	6	30

				transfer station (transport only)						
Infiltrating pit	21	Manual (no specialised equipment)	2	Wheels - machine-powered (transport only)	1			Aerobic FSM	6	30
Infiltrating pit	21	Human-powered with specialised equipment	2	Wheels - human-and/or machine-powered with transfer station (transport only)	1			Aerobic FSM	6	30
Infiltrating pit	21	Human-powered with specialised equipment	2	Wheels - machine-powered (transport only)	1			Aerobic FSM	6	30
Infiltrating pit	21					Wheels - human powered	2	Aerobic FSM	6	29
Infiltrating pit	21					Wheels - human-and/or machine-powered with transfer station	1	Aerobic FSM	6	28
Infiltrating pit	21	Manual (no specialised equipment)	2	Wheels - human-and/or machine-powered with transfer station (transport only)	1			Anaerobic FSM	3	27
Infiltrating pit	21	Manual (no specialised equipment)	2	Wheels - machine-powered (transport only)	1			Anaerobic FSM	3	27
Infiltrating pit	21	Human-powered with specialised equipment	2	Wheels - human-and/or machine-powered with transfer station (transport only)	1			Anaerobic FSM	3	27
Infiltrating pit	21	Human-powered with specialised equipment	2	Wheels - machine-powered (transport only)	1			Anaerobic FSM	3	27
Infiltrating pit	21					Wheels - human powered	2	Anaerobic FSM	3	26
Infiltrating pit	21					Wheels - human-and/or machine-powered with transfer station	1	Anaerobic FSM	3	25
Sealed tank with infiltration structure	***10***					***Wheels - machine powered***	***17***	***Aerobic FSM***	***6***	***33***
Sealed tank with	***10***					***Wheels - machine powered***	***17***	***Anaerobic FSM***	***3***	***30***

infiltration structure										
Sealed tank with infiltration structure	10	Human-powered with specialised equipment	2	Wheels - human- and/or machine-powered with transfer station (transport only)	1			Aerobic FSM	6	19
Sealed tank with infiltration structure	10	Human-powered with specialised equipment	2	Wheels - machine-powered (transport only)	1			Aerobic FSM	6	19
Sealed tank with infiltration structure	10					Wheels - human powered	2	Aerobic FSM	6	18
Sealed tank with infiltration structure	10					Wheels - human- and/or machine-powered with transfer station	1	Aerobic FSM	6	17
Sealed tank with infiltration structure	10	Human-powered with specialised equipment	2	Wheels - human- and/or machine-powered with transfer station (transport only)	1			Anaerobic FSM	3	16
Sealed tank with infiltration structure	10	Human-powered with specialised equipment	2	Wheels - machine-powered (transport only)	1			Anaerobic FSM	3	16
Sealed tank with infiltration structure	10					Wheels - human powered	2	Anaerobic FSM	3	15
Sealed tank with infiltration structure	10					Wheels - human- and/or machine-powered with transfer station	1	Anaerobic FSM	3	14
Sealed tank without infiltration structure	***9***					***Wheels - machine powered***	***17***	***Aerobic FSM***	***6***	***32***
Sealed tank without infiltration structure	***9***					***Wheels - machine powered***	***17***	***Anaerobic FSM***	***3***	***29***
Sealed tank without infiltration structure	9	Human-powered with specialised equipment	2	Wheels - human- and/or machine-powered with transfer station (transport only)	1			Aerobic FSM	6	18
Sealed tank without infiltration structure	9	Human-powered with	2	Wheels - machine-powered (transport only)	1			Aerobic FSM	6	18

		specialised equipment								
Sealed tank without infiltration structure	9					Wheels - human powered	2	Aerobic FSM	6	17
Sealed tank without infiltration structure	9					Wheels - human- and/or machine-powered with transfer station	1	Aerobic FSM	6	16
Sealed tank without infiltration structure	9	Human-powered with specialised equipment	2	Wheels - human- and/or machine-powered with transfer station (transport only)	1			Anaerobic FSM	3	15
Sealed tank without infiltration structure	9	Human-powered with specialised equipment	2	Wheels - machine-powered (transport only)	1			Anaerobic FSM	3	15
Sealed tank without infiltration structure	9					Wheels - human powered	2	Anaerobic FSM	3	14
Sealed tank without infiltration structure	9					Wheels - human- and/or machine-powered with transfer station	1	Anaerobic FSM	3	13
Direct	***8***					***Pipes - conventional, combined, with pumping***	***7***	***Machine-powered aerobic waste water***	***15***	***30***
Direct	***8***					***Pipes - conventional, separate, with pumping***	***6***	***Machine-powered aerobic waste water***	***15***	***29***
Direct	*8*					*Pipes - conventional, separate, no pumping*	*2*	*Machine-powered aerobic waste water*	*15*	*25*
Direct	***8***					***Pipes - conventional, combined, with pumping***	***7***	***Passive aerobic waste water***	***9***	***24***
Direct	***8***					***Pipes - conventional, separate, with pumping***	***6***	***Passive aerobic waste water***	***9***	***23***
Direct	*8*					*Pipes - conventional,*	*2*	*Passive aerobic*	*9*	*19*

			separate, no pumping		*waste water*		
Direct	8		Pipes - conventional, combined, with pumping	7	Anaerobic wastewater	1	16
Direct	8		Pipes - conventional, separate, with pumping	6	Anaerobic wastewater	1	15
Direct	8		Pipes - conventional, separate, no pumping	2	Anaerobic wastewater	1	11

Table S-3 Distribution of data points available in the CACTUS database by component and country as of June 2023.

		Bangladesh	China	Ghana	Guyana	India	Kenya	Peru	Senegal	Thailand	Zambia	Total
	**Containment**	**8**		**2**	**2**		**34**		**2**		**2**	**50**
	Container			1			1					2
	Direct	1		1	1		3		1		1	8
	Infiltrating pit	2					18				1	21
	Sealed tank with infiltration structure	1					9					10
	Sealed tank without infiltration structure	4			1		3		1			9
	**Emptying**	**1**		**1**			**2**					**4**
	Human-powered with specialised equipment						2					2
	Manual (no specialised equipment)	1		1								2
	**Emptying and Transport**	**8**		**1**	**2**	**2**	**9**	**6**	**2**	**1**	**4**	**35**
Pipes (sewers)	Conventional, combined, pumped					1		6				7
	Conventional, separate, no pumping						2					2
	Conventional, separate, pumped	1		1	1		1		1		1	6
Wheels (trucks)	Human- and/or machine-powered with transfer station						1					1
	Human-powered						1				1	2
	Machine-powered	7			1	1	4		1	1	2	17
	**Transport**			**1**			**1**					**2**
Wheels (trucks)	Wheels - human- and/or machine-powered with transfer station (transport only)						1					1
	Wheels - machine-powered (transport only)			1								1
	**Treatment**	**4**	**10**	**2**		**2**	**5**	**6**	**2**	**1**	**2**	**34**
	Aerobic FSM	3		1			1		1			6
	Anaerobic FSM					1				1	1	3
	Anaerobic wastewater						1					1
	Machine-powered aerobic waste water		10			1	2		1		1	15
	Passive aerobic waste water			1			1	6				8
	Passive aerobic wastewater	1										1
	**Total**	**21**	**10**	**7**	**4**	**4**	**51**	**12**	**6**	**2**	**8**	**125**

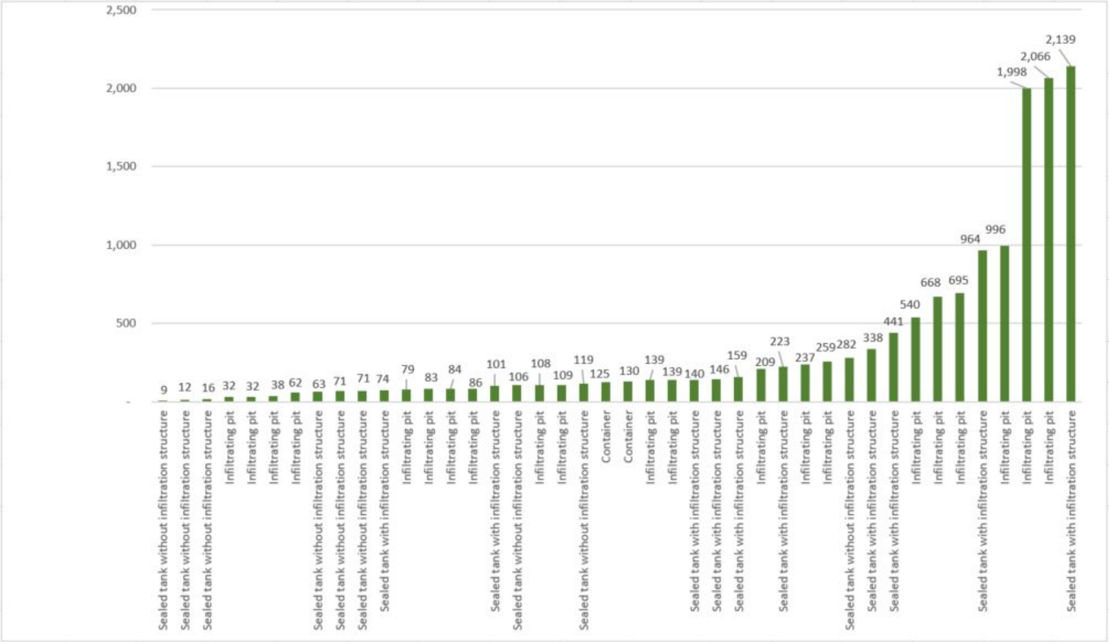

Panel A: Containers for onsite sanitation

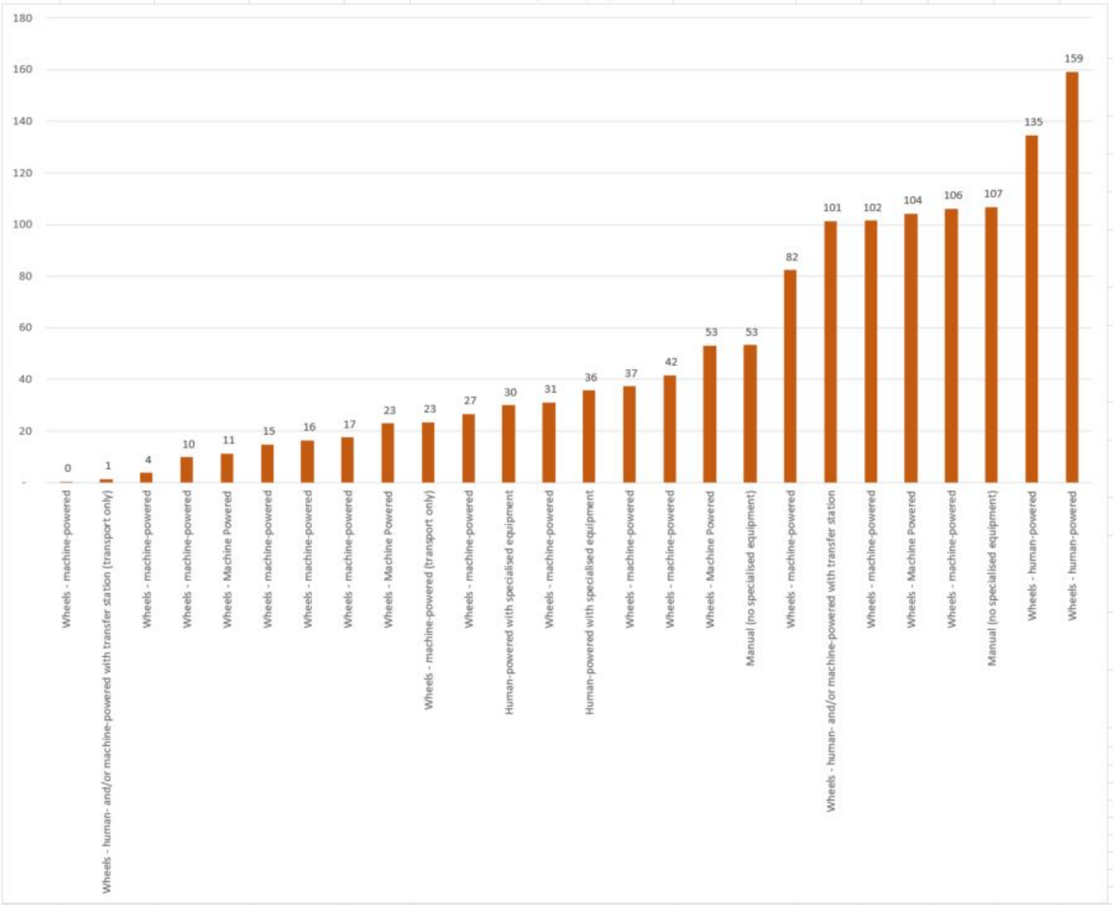

Panel B: Emptying, emptying and transport, and transport services

Figure S-2: Ranked total annualised cost liability (Int$2020) for containers and emptying/ transport (detail of specific data points) in urban sanitation systems with no sewerage – from data collected by the CACTUS Project as of February 2023.

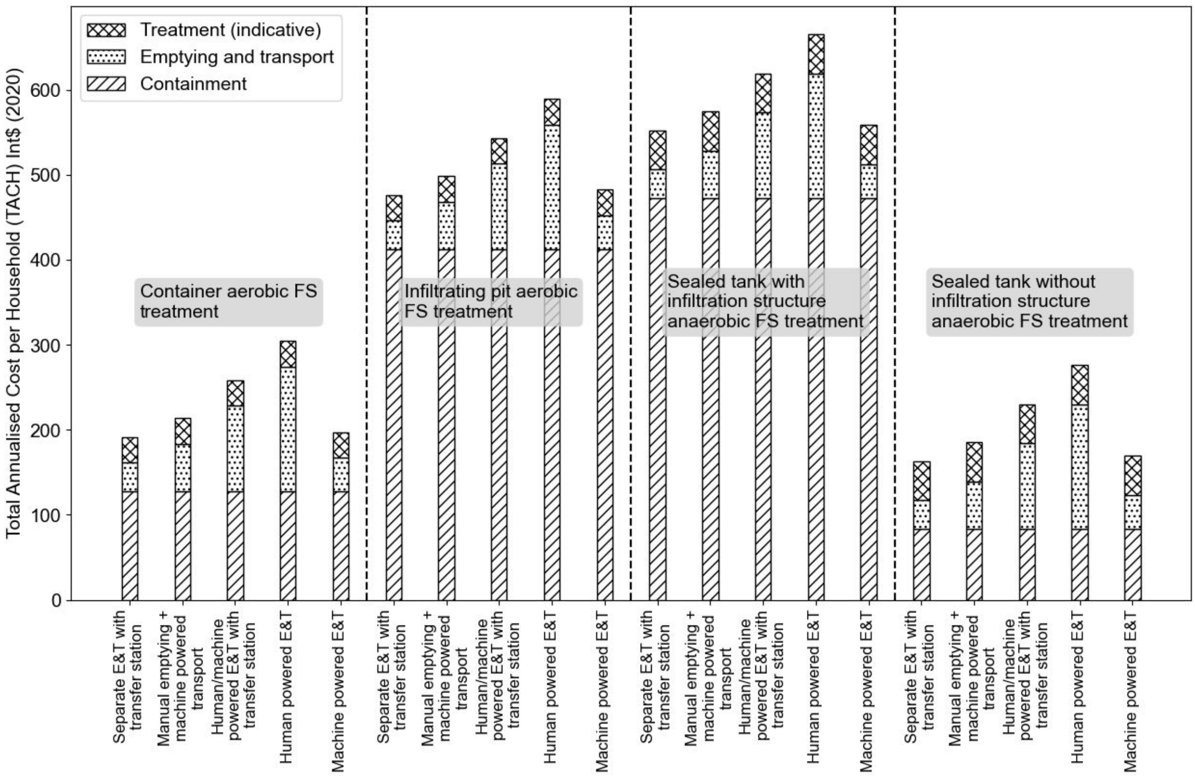

Figure S-3 Total annualised cost liability (Int$2020) modelled from mean data for non-sewered urban sanitation systems broken down by component from data collected by the CACTUS Project as of June2023.

Table S-4: Summary urban sanitation cost data from Narok Town (2022)

Component (n)	Description/ means of estimation	Total annualised cost Int$ (2020) Median, *Mean*, (Range) per household (TACH)	per capita (TACC)
Direct connection (1)	Comprises a series of inspection chambers with person access point and 160mm ø uPVC sewer	179	45
Sewer (1)	Conventional, separate sewers (10.8km of main trunk sewer, 14km of trunk sewer and 28km of secondary sewer) with no pumping (Running at 6% design capacity)	5,697	1,424
Infiltrating pit (9)	The typical design is that there are two toilets and two bathrooms on the top of a pit (1m × 2m × 6m depth)	188, *512*, (32 – 2066)	70, *133*, (5-666)
Sealed tank with infiltration (16)	The typical design is that a septic tank (3m × 6m × 3m depth) with a soak pit for infiltration which is connected to the toilets	*223, 517* (101-2139)	51, *129* (34-535)
Mechanical emptying and transport (2)	A vacuum truck to emptying human waste to transport to the treatment	-,- (11-104)	-,- (4-26)
Treatment (1)	Anaerobic treatment (four anaerobic ponds, two facultative ponds, 6 maturation ponds and a sludge drying bed)	146	36

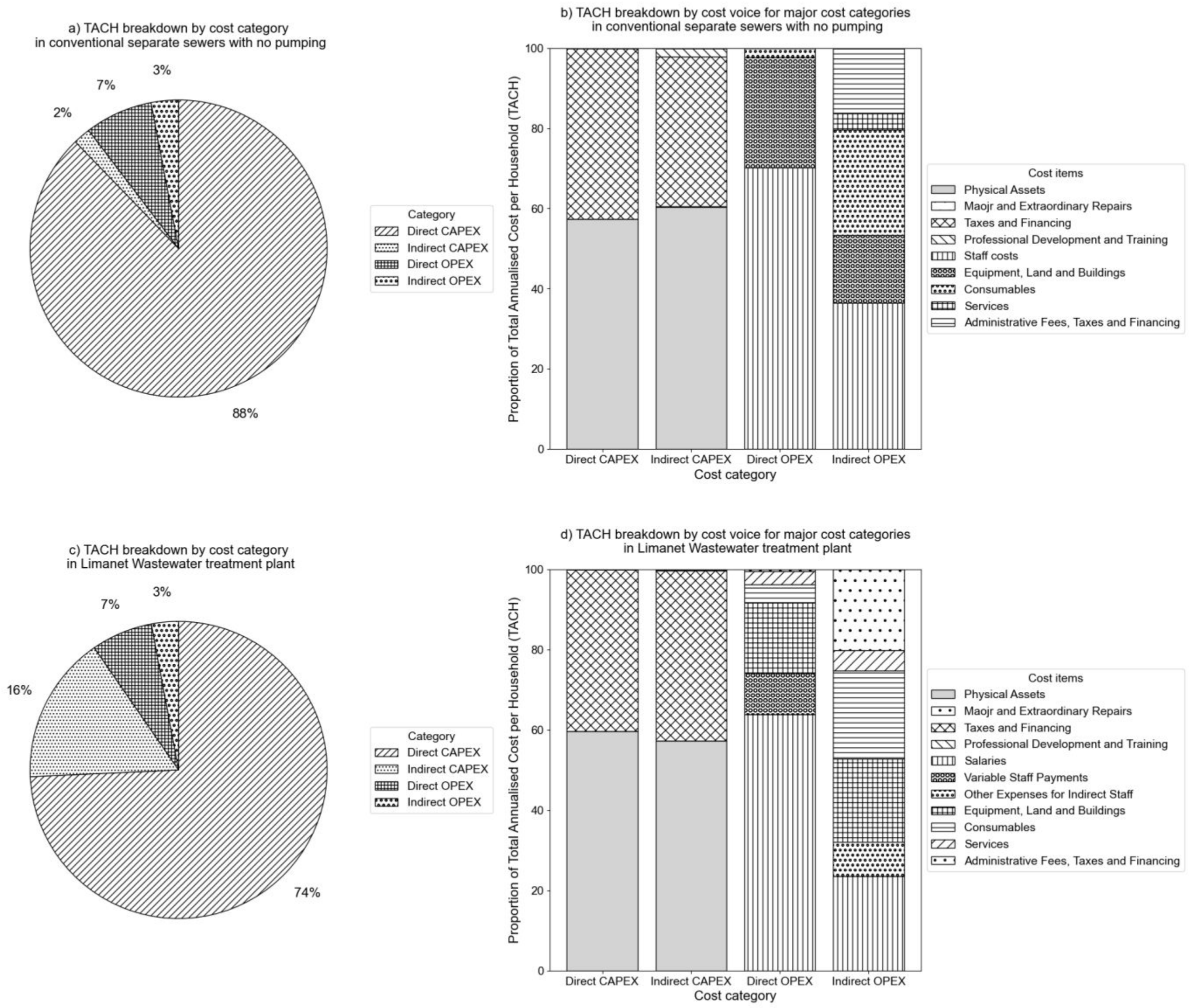

Figure S-4 Distribution of cost liability for sewerage (a and b) and wastewater treatment (c and d) in Narok town Kenya expressed in Total Annualised Cost per Household (TACH) as recorded in the CACTUS data base June2023. Capital costs are covered from the proceeds of a loan from the African Development Bank (AfDB). The loan has an interest rate of 3.5% and a repayment period of 30 years.

Notes on Figure S-4: The CACTUS database allows for further interrogation of data for a specific data point to understand cost drivers. For the sewer system in Narok 88% of the total cost liability per household (Int$ (2020) 5,107) is Direct CAPEX, of which 43% is the financing cost, and the remaining 57% is the direct cost of physical assets. Seventy percent of OPEX is staff costs (Figure S-4 panel a and b). For the Wastewater treatment plant, 74% of the total cost liability is Direct CAPEX of which 43% is taxes and financing costs. OPEX is dominated by expenses for staff (74% in direct and 32% in indirect) (Figure S-4 panel c and d).

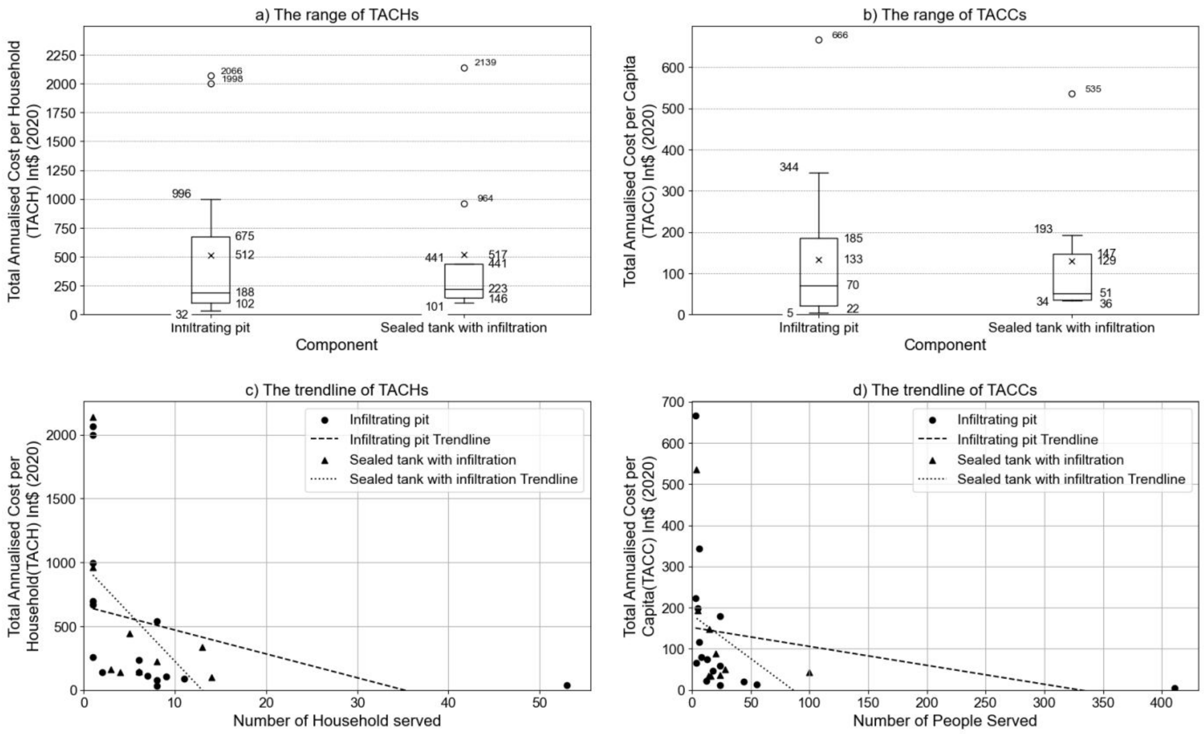

Figure S-5: Total Annualised Cost of onsite sanitation containments in Narok Town (Int$ 2020) (a) per household (TACH) (b) per capita (TACC), and the trendline of toilet cost against the number of households served (c) and the number of people served.

Notes on Figure S-5: TACH for onsite containment in Narok is largely in line with the global dataset although there are some much more costly systems, often within private households. Onsite containment costs per household in Narok are broadly inversely related to the numbers of household or people served (Figure S-5). There are some outliers, with significantly higher TACH/TACC - these are often extremely large and well-built and used by individual private households.

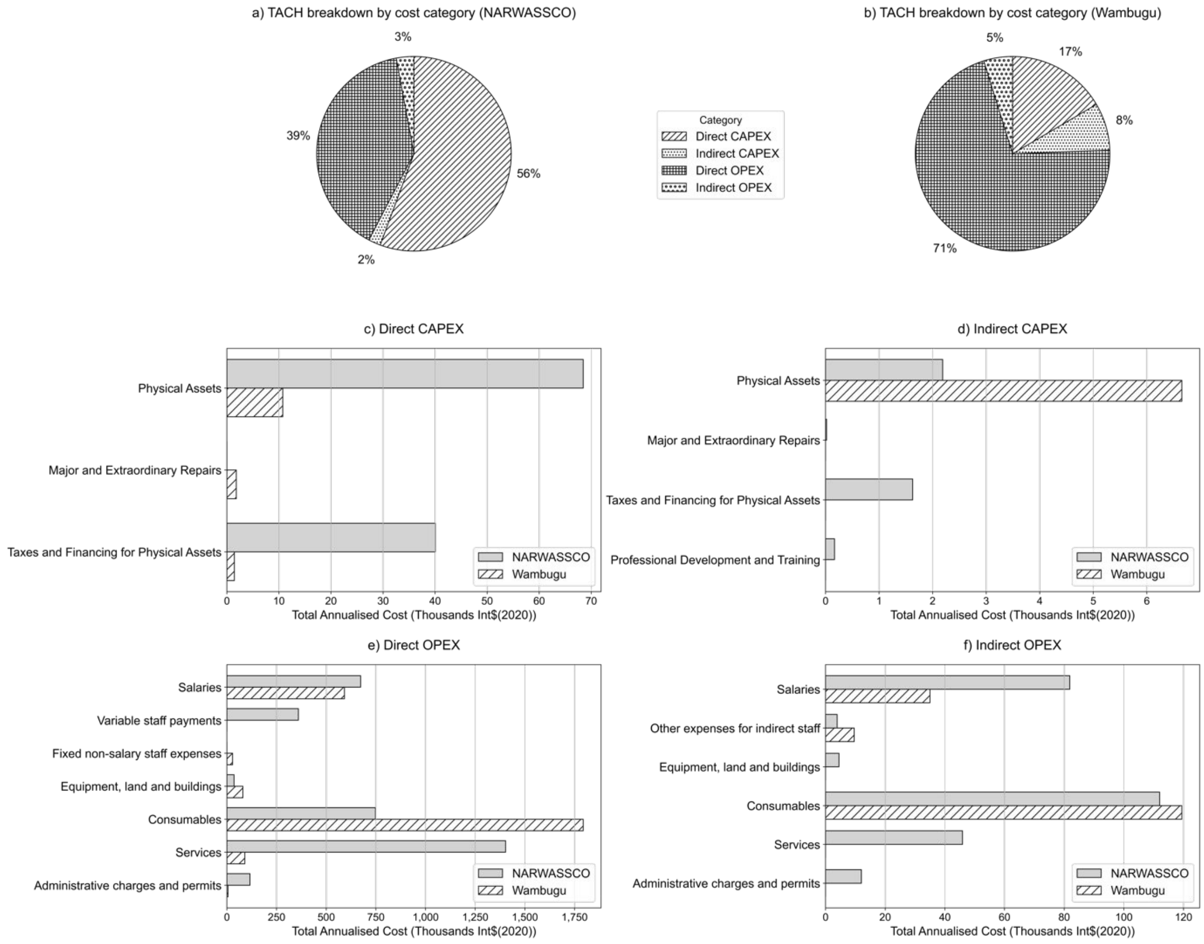

Figure S-6 Total annualised cost of mechanical emptying and transport for two providers in Narok Town, NARWASSCO (publicly owned) and Wambugu (privately owned). Plates a) and b) show cost breakdown for each major cost category. Cost breakdowns by subcategory are shown in plates (c) Direct CAPEX, (d) Indirect CAPEX, (e), Direct OPEX and (f) Indirect OPEX

Notes on Figure S-6: NARWASSCO has two trucks, compared to Wambugu's one, but serves a much smaller area (population of 7,488 compared to Wambugu's 22,680). NARWASSCO reported typically 6 households in a plot, while Wambugu reported generally 14 households. The results show the difference in cost efficiency of emptying and transport services between both operators. More efficient use of assets could improve the cost efficiency of NARWASSCO's operation. Here too, financing arrangements influence total costs. Both providers used loans to procure physical assets (trucks), however, the proportion of financing costs of CAPEX for NARWASSCO is 30% higher than for Wambugu. NARWASSCO used the loan from AfDB which financed the sewer system to also purchase new trucks for emptying and transport. By contrast, the private operator assembled one of their trucks second-hand. Operational costs for Wambugu are higher than for NARWASSCO

and are dominated by fuel costs reflecting the higher number of trips made per household served.
